# Supplementary material for: Design process and preliminary psychometric study of a video game to detect cognitive impairment in senior adults
Source: PeerJ. 2017 Jun 30;5:e3508. doi: 10.7717/peerj.3508 (PMC5494179; doi:10.7717/peerj.3508)
Supplement: Supplemental Information 1 [file peerj-05-3508-s001.pdf]

COGNITIVE EPISODIC GAME

Early prediction of MCI and AD. Design process and preliminary pyschometric study.

PeerJ, 2017

1. INTRODUCTION

- Episodic memory
- Early cognitive markers
- Classical tests limitations

Can we design a Digital Game to early diagnose MCI and AD?

MATERIALS & METHOD .2

- Gamification of CVLT
- More ecological tasks (i.e., virtual walk small city)
- User-centered design
- 2 Focus groups
- 1st Pilot estudy

3. RESULTS

- New word lists: A, B and C (i.e., furniture, shops, vehicles...)
- cognitive prediction using: Linear Regression, Random Forest & Support Vector Machine
- Precision=1 (LR, RF) and 0.90 (SVM)

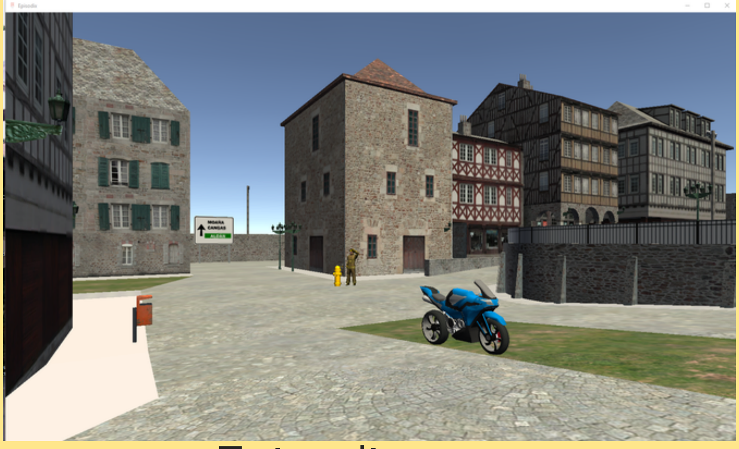

Episodix game

DISCUSSION .4

+ New scenarios closer to real life
+ More engaging than CVLT
+ High acceptability by end users
+ Initial promising results detecting cognitive problems

- To improve accesibility
- To test with a larger sample

CONCLUSIONS

- Seniors accept using games for early detection
- Cognitive problems correctly detected: promising results
- More reasearch is needed to provide clinical evidence
